# A case of H-type double gallbladder anomaly: A case report

**DOI:** 10.1097/MD.0000000000039284

**Published:** 2024-08-30

**Authors:** Yingxue Cheng, Zhe Xi, Aobo Zhuang, Xi Li, Yue Wang, Guangting Yan, Geng Zhang, Chenhe Zhang, Kun Chen, Yang Yang Huang, Changsheng Zhou, Qing Wang, Wengang Li

**Affiliations:** a Cancer Research Center, School of Medicine, Xiamen University, Xiamen, Fujian, PR China; b Xiamen University Research Center of Retroperitoneal Tumor Committee of Oncology Society of Chinese Medical Association, Xiamen University, Xiamen, Fujian, PR China; c Department of Biostatistics, School of Public Health, Harvard University, Cambridge, MA.; d Department of Hepatobiliary Surgery, Xiang’an Hospital of Xiamen University, School of Medicine, Xiamen University, Xiamen, Fujian, PR China

**Keywords:** case report, double gallbladder, gallbladder malformation, treatment

## Abstract

**Rationale:**

Biliary system anomalies, such as duplicated gallbladders, are rare congenital conditions that present significant diagnostic challenges. Dr. Boyden’s classification system, especially the H-type anomaly, offers vital insight into these variations. Failure to detect these anomalies preoperatively can increase the risk of surgical complications, making early identification crucial for surgical planning.

**Patient concern:**

A 42-year-old male, asymptomatic, was incidentally found to have a gallbladder mass during routine imaging. An upper abdominal magnetic resonance imaging showed gallbladder wall thickening, gallstones, and a liver lesion. Despite the absence of symptoms, a laparoscopic cholecystectomy revealed an atrophied gallbladder with a cystic duct cyst, which was identified as an H-type double gallbladder anomaly. The surgery was completed without complications, and pathology confirmed the presence of gallstones and inflammation.

**Diagnoses:**

The patient was diagnosed with a duplicated gallbladder, classified as an H-type anomaly, following laparoscopic cholecystectomy. Preoperative imaging identified gallbladder wall thickening and gallstones, and further investigation during surgery confirmed the congenital anomaly.

**Interventions:**

The patient underwent laparoscopic cholecystectomy for the removal of the gallbladder, and during the procedure, an H-type double gallbladder anomaly was discovered. The surgery proceeded without incident, ensuring the complete excision of the gallbladders.

**Outcomes:**

The case highlights the diagnostic difficulty of identifying duplicated gallbladders and the importance of advanced imaging techniques in detecting atypical anatomical variations. The successful laparoscopic removal of both gallbladders illustrates the current capabilities of minimally invasive surgery. Postoperative recovery was uneventful, and the pathology confirmed gallstones and inflammation.

**Lessons:**

This case emphasizes the importance of recognizing biliary anomalies such as duplicated gallbladders to avoid complications during surgery. Preoperative identification, aided by imaging, and careful surgical planning are key to managing these rare conditions. The case contributes to the growing body of knowledge about biliary system anomalies and reinforces the need for comprehensive management strategies to ensure optimal patient outcomes.

## 1. Introduction

Anomalies within the biliary system are not an uncommon occurrence, often involving the bile ducts or gallbladder, such as biliary atresia, duplicated gallbladder, and Luschka ducts. Among these, a duplicated gallbladder stands out as a rare congenital anomaly with an incidence of 1 in 4000.^[1]^ Its diagnosis is typically elusive through routine ultrasound examinations, posing a challenge for clinicians to make a definitive preoperative assessment.^[2]^ Failure to identify a duplicated gallbladder before or during surgery may result in unintended damage to the bile ducts and blood vessels during the procedure.^[3]^

Dr Boyden pioneering work involved reporting the morphological variations of double gallbladders through meticulous cadaver dissections and radiological investigations. He classified this duplication into 3 types: Type I, featuring separated gallbladders with internal communication via a common cystic duct; Type II, presenting Y-shaped double gallbladders without internal communication, each with an independent cystic duct, merging before joining the common bile duct; Type III, characterized by an H-shaped double gallbladder configuration with 2 independent gallbladders, each having its distinct cystic duct, and both cystic ducts separately joining the common bile duct. Among these classifications, the H-type anomaly is the most frequently encountered in cases of duplicated gallbladders.^[4]^ Currently, double gallbladder anomaly remains a highly rare condition, so we are discussing a case treated at our hospital regarding this disease.

## 2. Case

**Figure 1.**
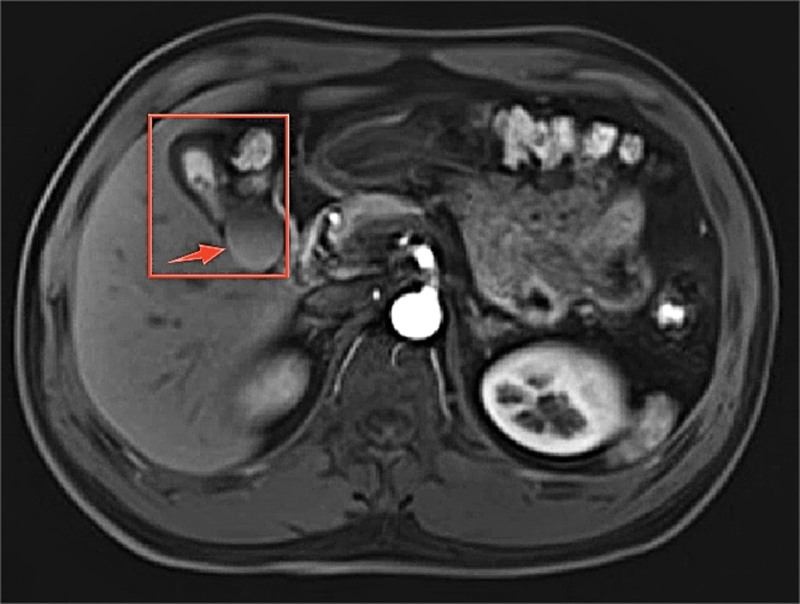
Preoperative MRI scan of the patient’s upper abdomen. The area within the red box is the cystohepatic triangle; the red arrow points to the cystic mass. MRI = magnetic resonance imaging.

The patient is a 42-year-old male who, during a routine health checkup at another hospital 1 day prior, incidentally discovered a cystic mass around the gallbladder via ultrasound. He was subsequently transferred to our hospital, where an abdominal magnetic resonance imaging (MRI) scan (Fig. 1) performed on January 2024 revealed a thickened gallbladder wall with an adjacent cystic lesion, raising concerns about malignancy. Additionally, gallstones were observed, along with a nodular lesion in the S4 segment of the liver lacking adequate blood supply. Notably, the patient reported no abdominal pain, fever, nausea, vomiting, or diarrhea, prompting his decision to seek further diagnosis and treatment at our hospital. Throughout the course of the illness, the patient maintained normal mental well-being, dietary habits, sleep patterns, and regular urination and bowel movements. There were no discernible changes in physical strength or weight.

Upon admission, the abdominal examination unveiled tenderness in the flat abdomen, with a soft and non-tender abdomen, devoid of palpable masses in the liver or spleen. The gallbladder displayed no overt abnormalities, and Murphy sign elicited a negative response. Percussion over the abdomen resulted in a resonant sound, and there was an absence of shifting dullness. Bowel sounds were within the normal range. After ruling out contraindications for surgery, a laparoscopic cholecystectomy was performed under general anesthesia on January 9, 2024.

**Figure 2.**
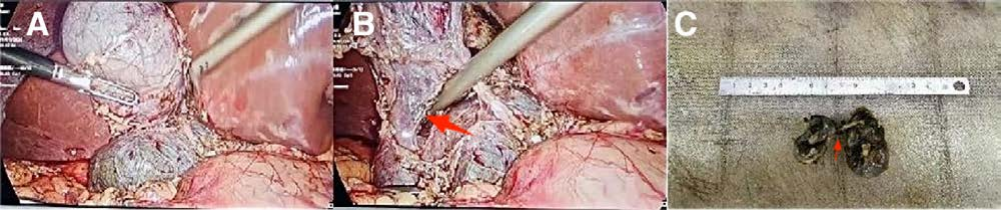
Operation diagram of laparoscopic double gallbladder malformation resection. (A) Double gallbladder structure was seen during operation; (B) the connecting duct between the double gallbladder can be seen intraoperatively; (C) both gallbladder were completely free after surgery. The red arrow points to the duct between the H-type double gallbladder anomaly.

Intraoperatively, significant adhesions were encountered in the gallbladder bed omentum. Upon meticulous separation of these adhesions, the gallbladder was found to be atrophied, measuring approximately 3 × 2 cm. A cystic mass on the inner side of the gallbladder, measuring about 4 × 5 cm, was identified, suggesting a possible cystic duct cyst (Fig. 2A). Manipulating the gallbladder revealed adhesions of the gallbladder artery, which traversed the cystic duct cyst. Further dissection uncovered a communication channel between the gallbladder and the cyst (Fig. 2B). After free dissection on the right side of the common bile duct and fine-needle aspiration for duct localization, it was observed that there was a cystic duct between the cystic duct cyst and the common bile duct, confirming the presence of a double gallbladder anomaly. The surgical procedure proceeded seamlessly without any bile duct damage, and there was no need for a conversion to open surgery. Postoperative pathological analysis indicated the presence of gallstones concurrent with gallbladder inflammation and confirmed the existence of a double gallbladder anomaly (Fig. 2C). The duration of the surgery was 2 hours, with an intraoperative blood loss of 20 mL. Notably, the patient did not experience any postoperative complications and was discharged 3 days later.

## 3. Discussion

The presented case of a duplicated gallbladder anomaly offers valuable insights into the challenges and clinical management of this rare congenital condition. Duplicated gallbladders, although uncommon, belong to a spectrum of anomalies within the biliary system, and their diagnosis poses a significant challenge due to the elusive nature of detection through routine ultrasound examinations.

The case study involving a 42-year-old male with incidental findings of a gallbladder mass highlights the complexity of diagnosing duplicated gallbladders, especially considering the absence of typical symptoms. The use of advanced imaging techniques, such as upper abdominal MRI scans, played a crucial role in revealing thickening of the gallbladder wall, gallstones, and a nodular lesion in the liver’s S4 segment. The successful laparoscopic cholecystectomy, performed after meticulous exclusion of surgical contraindications, showcased the contemporary advancements in the surgical management of gallbladder anomalies. The intraoperative discovery of significant adhesions, atrophied gallbladder, and a cystic duct cyst demonstrated the intricacies associated with double gallbladder anomalies. The absence of bile duct damage and the seamless surgical procedure underscore the efficacy and safety of laparoscopic techniques.

By searching databases including Wanfang, China national knowledge infrastructure (CNKI), PubMed, Web of Science, and others, we discovered that among 18 cases of patients with H-type double gallbladder anomaly, the male-to-female ratio was 4:5.^[5]^ While gallbladder diseases tend to have a higher incidence in females, studies also suggest that the occurrence of double gallbladder anomalies is comparable between genders.^[2]^ Embryologically, double gallbladders originate from the distal part of the hepatic diverticulum, and abnormal differentiation of the primitive gallbladder during the 4th and 5th weeks of pregnancy may result in the development of multiple gallbladders.^[5]^ From a surgical standpoint, when surgeons encounter an accessory gallbladder during the procedure, it is advisable to remove both gallbladders simultaneously to prevent potential complications in the future.^[6–8]^ Failure to remove the accessory gallbladder may lead to the manifestation of biliary symptoms.^[9–11]^ The accessory gallbladder is susceptible to all the same diseases as the primary gallbladder, including cholecystitis, gallbladder-colon fistula, cholelithiasis, and gallbladder cancer.^[12–15]^ Therefore, in order to prevent the recurrence of biliary symptoms and the emergence of new biliary issues, we believe that identifying and excising the accessory gallbladder during surgery is essential.

In conclusion, this case study not only contributes to the understanding of duplicated gallbladder anomalies but also reflects the evolving landscape of diagnostic and surgical approaches in contemporary clinical practice. Heightened awareness, advanced imaging technologies, and precise surgical interventions collectively enhance the management of rare biliary anomalies, ensuring optimal patient outcomes.

## Author contributions

**Writing – original draft:** Yingxue Cheng, Zhe Xi, Aobo Zhuang, Xi Li, Yue Wang, Guangting Yan, Geng Zhang, Chenhe Zhang, Kun Chen, Yangyang Huang.

**Writing – review & editing:** Changsheng Zhou, Qing Wang, Wengang Li.
